# Challenges associated with sexually transmitted infection partner notification approaches in Africa: a scoping review protocol

**DOI:** 10.3389/frph.2026.1951049

**Published:** 2026-09-15

**Authors:** Noluthando Mpobole, Raikane James Seretlo, Keatlegile Moses Eskia Mabena, Hanlie Smuts, Mathildah Mpata Mokgatle

**Affiliations:** 1 Department of Public Health, School of Healthcare Sciences, Sefako Makgatho Health Sciences University Ga-Rankuwa Pretoria, Pretoria, South Africa; 2 Department of Development Studies, College of Human Sciences, University of South Africa, Pretoria, South Africa; 3 Department of Informatics, Faculty of Built Environment, Engineering, and IT, University of Pretoria, Pretoria, South Africa

**Keywords:** Africa, health care providers, partner notification, public health, sexually tranmistted infections

## Abstract

**Background:**

Despite extensive prevention efforts, sexually transmitted infections (STIs) remain a major public health concern across Africa. Partner notification (PN) is an important component of STI case management that aims to identify and manage sexual partners of people diagnosed with STIs and thereby reduce onward transmission and reinfection. Previous reviews have examined PN strategies in developing countries and sub-Saharan Africa, including acceptability, efficacy, barriers and facilitators, but these reviews have focused on specific populations, outcomes or periods and have not comprehensively mapped challenges experienced by both patients and healthcare providers across African settings. This review will therefore map the nature, range and distribution of reported challenges associated with current STI PN approaches across Africa.

**Objective:**

The objective of this scoping review is to map out the challenges experienced by healthcare providers and patients with current STI PN approaches across Africa.

**Methods and analysis:**

Guided by the Joanna Briggs Institute (JBI) methodology for scoping reviews and the Population-Concept-Context(PCC) framework, a systematic search will be conducted across PubMed, Scopus, Web of Science CINAHL and African Journals Online (AJOL), supplemented by Google Scholar, and specified grey literature sources. Peer-reviewed qualitative, quantitative and mixed-methods studies published in English between 2011 and 2026 focusing on STI PN challenges in African settings will be included. Two reviewers will independently screen titles, abstracts and full texts. Two reviewers will independently screen titles, abstracts and full texts and chart data using a standardised JBI-informed charting form. Disagreements will be resolved through consensus or adjudication by a third reviewer. The results will be synthesized narratively and presented using tables and thematic maps.

**Discussion:**

By systematically mapping structural, systemic and interpersonal barriers such as stigma, fear of violence, resource constraints and linguistic limitations this review will highlight critical knowledge gaps in STI management in Africa.

## 1 Introduction

Every untreated sexual partner represents a missed opportunity to interrupt the transmission of sexually transmitted infections (STIs). STIs remain a major public health concern globally and across Africa. Despite decades of STI prevention efforts, the burden of STIs remains high across Africa. An overall STI prevalence of 74.4% was reported among patients attending healthcare facilities in Ethiopia (1). In South Africa, 37% of pregnant women attending their first antenatal visit were diagnosed with STIs (2), while approximately 40% of men attending public health facilities in Gauteng Province in 2023 were treated for male urethritis syndrome (3). Partner notification (PN) is a foundational public health service designed to identify and inform sexual partners of their potential exposure to an STI and encourage them to undergo STI screening and treatment (4). PN can help identify undiagnosed infections, prevent reinfection and interrupt chains of transmission. PN aims to reduce the prevalence of infections such as chlamydia, syphilis and Human Immune Deficiency Virus (HIV) (5). Most STIs are asymptomatic and PN is important for preventing re-infection, curbing transmission and contributes to improved sexual and reproductive health outcomes (6).

Various African countries use different PN interventions to ensure prevention and the control of STIs. For instance, South African clinical guidelines suggest that for the treatment and management of STI, every person who tests positive should be given a notification slip to give to their partners (7). Ferreiea et al (8), identified several mechanisms for PN, including patient referral, provider referral and expedited partner therapy. In provider referral, healthcare providers directly notify the sexual partners of index patients and facilitate access to testing and treatment. Other approaches include mixed or two-step strategies, in which patients are first encouraged to notify their partners themselves, with healthcare providers undertaking notification when patient referral is unsuccessful (9). PN and treatment rates are still poor despite these recommendations and research carried out in African countries has revealed a variety of obstacles impacting the adoption and use of STI PN. Many adolescent girls and young women (AGYW) accept the PN STI slip without intending to notify their partner, either due to fear or lack of communication skills about STIs (10). When informing partners, patients often worry about intimate partner violence, relationship conflict, stigma, blame, rejection and confidentiality (11). Furthermore, healthcare providers similarly face challenges, limited resources, staff shortages, heavy workloads, insufficient follow-up mechanisms and the absence of standardized PN systems (12).

The failure to address problems with the current STI PN methods may prolong the spread of STIs, raise the risk of reinfection among those receiving treatment, delay the diagnosis and treatment of exposed partners and worsen negative outcomes related to sexual and reproductive health. Furthermore, inadequate PN may result in untreated STIs. Untreated STIs can lead to recurrent or chronic infections and serious health complications. Women are particularly at risk of chronic pelvic pain, pelvic inflammatory disease and infertility (13). Untreated STIs also increase the risk of HIV acquisition, especially among sexually active young people (14). In addition, they may result in reduced sexual function, low self-esteem, infertility and mortality (15).

Previous evidence syntheses have examined PN strategies in developing countries and sub-Saharan Africa, including acceptability, efficacy, barriers and facilitators (16, 17). However, these reviews differ from the present protocol in scope and emphasis. The earlier developing-country review included evidence published up to 2007 and examined feasibility, acceptability, outcomes and barriers; a later systematic review focused specifically on curable STIs in sub-Saharan Africa and examined acceptability, efficacy, barriers and facilitators; and a recent 2026 scoping review focused on youth experiences and practices in sub-Saharan Africa (18). None of these reviews was designed to comprehensively map challenges experienced by both patients and healthcare providers across all African regions and across the range of current STI PN approaches. This scoping review aims to map out the challenges experienced by healthcare providers and patients in the implementation and use of current STI PN approaches in Africa. The findings seeks to provide an overview of existing evidence, identify knowledge gaps and inform future research, policy and intervention development aimed at strengthening STI PN practices in African settings. This scoping review forms Phase 1 of a larger study aimed at developing adigital PN system to inform sexual partners of STI exposure. The findings from this review will inform the subsequent development phase by identifying the challenges and opportunities associated with existing STI PN approaches. The review team will apply the predefined eligibility criteria and data-charting framework consistently, and interpretation will remain focused on the evidence reported in the included studies rather than on the potential usefulness of digital solutions.

## 2 Materials and methods

### 2.1 Population

This review will focus on patients diagnosed with STIs and healthcare providers involved in STI PN services across Africa. Healthcare providers may include nurses, doctors, counsellors, community health workers, health promoters and other professionals responsible for STI management and PN activities.

### 2.2 Concept

The concept of this scoping review is the challenges or barriers associated with the implementation and use of STI PN approaches. Challenges may occur at individual, interpersonal, provider, health-system, organisational, sociocultural, structural, policy, linguistic or technological levels. The review will primarily map barriers and challenges; facilitators will be charted when reported to provide context and contrast. STI PN approaches will include patient referral, provider referral, contract referral, expedited partner therapy, mixed or dual referral strategies, and digital or anonymous approaches used to notify or support notification of sexual partners.

### 2.3 Context

The context is African countries and healthcare or community settings in which STI PN is implemented or experienced. Africa will be interpreted as including both North African and sub-Saharan African countries. Studies conducted in facility-based, community-based, outreach, primary healthcare, specialist or other relevant STI service settings will be eligible. Country and African sub-region will be charted to allow comparison of evidence across geographic contexts.

### 2.4 Study design

This scoping review will be conducted in accordance with the JBI methodology for scoping reviews. The JBI framework provides a systematic and transparent approach for identifying, mapping and synthesising evidence across a broad range of study designs. The review will follow the five-stage framework proposed by (19), which are identifying the research question, identifying relevant studies, selecting eligible studies, charting the data and collating, summarising and reporting the results. The review will be reported according to the Preferred Reporting Items for Systematic Reviews and Meta-Analyses Extension for Scoping Reviews (PRISMA-ScR) (20). The study is registered at Open Science Framework (https://doi.org/10.17605/OSF.IO/DSXBC).

### 2.5 Review question

The scoping review, guided by the PCC framework aims to map the challenges associated with current STI PN approaches as experienced by healthcare providers and patients in Africa. The review question is: what are challenges that are experienced by healthcare providers and patients with current STI PN approaches across Africa? The review will consider challenges occurring at individual, interpersonal, healthcare-provider, health-system, organisational, sociocultural, structural, policy, linguistic and technological levels.

### 2.6 Search strategy

A comprehensive search strategy will be developed to identify relevant studies. A preliminary search of PubMed will be conducted to identify relevant keywords, synonyms and index terms related to STIs, PN, barriers, challenges, hinders, obstacles, difficulties, healthcare providers, healthcare workers, clinicians, service providers, care providers, medical practitioners, patients, service users, health service users, care recipients, African countries and regions. The search will combine controlled vocabulary (including MeSH terms in PubMed). The search terms will include, but will not be limited to, terms related to STIs and STDs, PN, patient referral, provider referral, contract referral, expedited partner therapy, digital PN, anonymous PN, barriers, challenges, obstacles, difficulties, stigma, confidentiality, healthcare providers, healthcare workers, clinicians, nurses, counsellors, community health workers, patients and service users. These identified keywords and index terms will be used to develop a comprehensive search strategy tailored to each database. Boolean operators (“AND” and “OR”) will be used to combine search terms where appropriate. The search strategy developed for PubMed will subsequently be adapted to the indexing requirements of each database. The databases to be searched will include PubMed, Scopus, Web of Science, CINAHL and African Journals Online (AJOL). Google Scholar will also be searched as a supplementary source, and the first 200 results ranked by relevance will be screened for potential eligibility. The search will cover studies published from 2011 to 2026. The complete database-specific search strategies, including the search terms, Boolean operators and database-specific subject headings, will be provided in Supplementary Table S3 to facilitate reproducibility. The reference lists of all included studies and relevant review articles will also be screened to identify additional potentially eligible studies. Grey literature will be searched through relevant organisational and institutional sources, including international health organisations, national health departments, institutional repositories and repositories containing theses and dissertations. The specific grey literature sources and corresponding search approaches will be reported in the supplementary search strategy.

### 2.7 Information sources

A comprehensive search will then be conducted in PubMed, Scopus, Web of Science Searches will be conducted in PubMed, Scopus, Web of Science, CINAHL and African Journals Online (AJOL), supplemented by Google Scholar. Grey literature will be searched through specified sources, including WHO Institutional Repository for Information Sharing (IRIS), ProQuest dissertations and theses where accessible, relevant national Departments of Health repositories and institutional repositories. Reference lists of all included studies and relevant reviews will also be screened for additional studies. For Google Scholar, the first 200 results ranked by relevance will be screened for feasibility. The search date for each database will be recorded and reported. The full search strategies and search dates will be provided.

### 2.8 Inclusion and exclusion criteria

The PCC framework will guide the selection of relevant studies. This review will include peer-reviewed studies published in English between 2011 and 2026 that focus on STI PN approaches in African countries, the review is intended to capture the most recent evidence available at the time of search. The 2011 start date was selected to focus the review on contemporary evidence on STI PN approaches following earlier evidence syntheses whose searches covered literature up to 2007, while allowing a sufficiently broad period to capture changes in PN delivery and emerging approaches. Eligible studies will report on the challenges or barriers experienced by healthcare providers and/or patients in the implementation or use of STI PN services. The review will consider studies involving patients diagnosed with STIs, healthcare providers involved in STI PN services or both. Qualitative, quantitative and mixed-methods study designs will be included to capture a comprehensive understanding of challenges related to STI PN. Studies conducted outside Africa, studies that do not report on challenges or barriers related to STI PN approaches and studies that do not involve the defined population will be excluded. Non-peer-reviewed publications, editorials, letters to the editor and conference abstracts without full reports will also be excluded (see Supplementary Table S1).

Supplementary Table S1 presents the inclusion and exclusion criteria according to the Population, Concept and Context (PCC) framework.

### 2.9 Study selection

All retrieved records will be imported into EndNote reference management software, where duplicates will be removed. The de-duplicated records will then be exported to Rayyan software for screening, a web-based systematic and scoping review screening platform designed to facilitate the efficient screening and selection of studies, for title, abstract and full-text screening. The reviewers will pilot the eligibility criteria on a sample of retrieved records to ensure consistent interpretation of inclusion and exclusion criteria. Two primary reviewers will independently screen all titles and abstracts against the predefined eligibility criteria. Records considered potentially eligible by either reviewer will proceed o full-text screening. Two primary reviewers will independently assess the full-text articles against eligibility criteria. Any disagreements that arise during the study selection will be resolved through discussion between the primary reviewers and the third reviewer. To enhance the rigor and trustworthiness of the review process, the remaining two supervisors will independently verify and validate the study selection decisions and procedures. The study selection process will be reported using a PRISMA-ScR flow diagram.

### 2.10 Data extraction

Data will be charted independently by two reviewers using a standardised JBI data-charting form. The form will be pilot tested on a sample of included studies before full extraction. The variables will include author(s), year of publication, country, African sub-region, study setting, study aim, study design, participant characteristics, age group, sex/gender where reported, STI or STI syndrome, PN approach type, reported challenges and barriers, facilitators where reported, key findings and recommendations. PN approach type will be operationalised according to the taxonomy described above, including patient referral, provider referral, contract referral, expedited partner therapy, mixed/dual referral and digital or anonymous approaches. Urban/rural setting and relevant population subgroups will also be charted where reported. The charting form may be refined iteratively; substantive amendments will be documented and agreed by the review team. Missing information will be recorded as “not reported.” Where clarification of essential information is required and feasible, corresponding authors may be contacted. The extracted data will be organised into tables and narrative summaries (see Supplementary Table S2).

Supplementary Table S2 presents the proposed data-charting variables that will be extracted from each included study.

### 2.11 Data synthesis and presentation

Findings will be synthesised narratively and mapped according to the review question and PCC framework. Descriptive characteristics of the included studies will be summarised in tables, including authorship, year, country, African sub-region, study design, setting, population and PN approach. Reported challenges will be organised thematically across individual, interpersonal, provider, health-system, organisational, sociocultural, structural, policy, linguistic and technological levels where supported by the evidence. The review will compare patterns across African subregions and PN approach types where sufficient information is available. Thematic maps will be used to visually illustrate recurring patterns and gaps. Facilitators will be reported when identified, but the primary synthesis will focus on challenges. The selection process will be presented using a PRISMA-ScR flow diagram (20). Findings will be used to inform the subsequent qualitative and development phases of the broader study without treating the scoping review as an evaluation of a proposed digital intervention.

## 3 Discussion

This protocol describes a scoping review designed to map the nature, range and distribution of challenges associated with STI PN approaches across African settings. A scoping review is appropriate because the evidence is expected to be heterogeneous in study design, population, setting and PN approach, and may be distributed across clinical, public health and social science literature. The review will provide a structured overview of the available evidence and identify areas where evidence is limited or fragmented.

The review is expected to clarify how challenges to STI PN are distributed across patient, interpersonal, provider, health-system, sociocultural, structural and technological levels. Although barriers such as stigma, fear of adverse partner reactions, intimate partner violence, confidentiality concerns, provider capacity and health-system constraints have been reported in previous literature, these will be treated as areas for examination rather than assumed findings. The review will assess how consistently such challenges are reported across African settings and PN approaches. The findings may inform STI programme planning, provider training, partner-management policies and the development of context-sensitive PN interventions. The findings will also inform subsequent qualitative and participatory phases of the broader study, including the assessment of whether and how digital approaches could address identified needs. Interpretation will remain evidence-led to minimise the risk that the broader digital intervention objective influences the thematic mapping.

## 4 Strengths and limitations

Strengths of this protocol include the use of a structured JBI methodology and PRISMA-ScR reporting framework, a broad PCC scope, inclusion of qualitative, quantitative and mixed-methods evidence, and searching multiple bibliographic and grey-literature sources. The review is designed to capture variation across African sub-regions, populations, health-system contexts and PN approaches. Several limitations are anticipated. First, restricting inclusion to English-language studies may underrepresent evidence from French-, Portuguese- and Arabic-speaking African settings and may introduce linguistic bias. Second, heterogeneity in study designs, populations, STI conditions and PN approaches may complicate comparison and thematic mapping. Third, although multiple databases and grey-literature sources will be searched, publication and retrieval bias may remain, particularly where relevant grey literature is not systematically indexed. Fourth, Google Scholar screening will be limited to the first 200 relevance-ranked results for feasibility, which may omit less highly ranked studies. Fifth, the literature may conflate STI PN with HIV status disclosure, contact tracing or other partner-services terminology, creating a risk of both false inclusion and omission; the search strategy and full-text screening will therefore be used to distinguish STI-specific PN evidence. Finally, where duplicate publications arise from the same underlying dataset, linked reports will be identified to reduce double-counting.
