# Supplementary material for: Challenges associated with sexually transmitted infection partner notification approaches in Africa: a scoping review protocol
Source: Front Reprod Health. 2026 Sep 15;8:1951049. doi: 10.3389/frph.2026.1951049 (PMC13619885; doi:10.3389/frph.2026.1951049)
Supplement: Supplementary file 1 [file Datasheet1.pdf]

Supplementary Material

1 Supplementary Tables

Table 1 Summary of PCC framework

PCC	INCLUSION	EXCLUSION
Population	Studies involving patients diagnosed with STIs, healthcare providers involved in STI PN services.	Studies that do not involve patients diagnosed with STIs, healthcare providers involved in STI PN services or both.
Concept	Studies examining STI PN approaches or strategies and reporting challenges, barriers, or negative experiences associated with their implementation or use. PN approaches may include patient referral, provider referral, contract referral, dual/mixed referral, expedited partner therapy, and digital or anonymous approaches.<br><br>Studies that report challenges related to STI PN.	Studies that do not focus on STI PN approaches or strategies<br><br>Studies that do not report challenges, barriers or experiences related to STI PN.
Context	Studies conducted in African countries, including North Africa and sub-Saharan Africa, across community, primary healthcare, hospital, STI clinic, antenatal and other relevant healthcare settings.	Studies conducted outside African countries.

2 Table 2 Data-charting form

Author,<br><br>year	Country/ specific location	African sub-region	Study setting	Study aim	Study design	Study population	Age/ group	STI/ condition	PN approach	Reported barriers	Key findings	Recommendations

3 Table 3 Planned database specific search strategy

Database	Search concepts	Keywords/ search terms	Search approach
PubMed	STI; PN; challenges/barriers; patients and healthcare providers; Africa	STI: “Sexually Transmitted Diseases”(MeSH) OR “sexually transmitted infection” OR “sexually transmitted disease” OR STI OR STD	Controlled vocabulary (MeSH) and free-text terms will be combined using AND/OR. The strategy will be adapted to PubMed syntax. English language; 2011–2026.
Scopus	STI; PN; challenges/barriers; patients and healthcare providers; Africa	Equivalent terms for STI/STD, partner notification/referral, barriers/challenges, patients, healthcare providers and African countries/regions	Free-text terms will be adapted to Scopus syntax using Boolean operators, truncation and phrase searching where appropriate. English language; 2011–2026.
Web of Science	STI; PN; challenges/barriers; patients and healthcare providers; Africa	Equivalent terms for STI/STD, partner notification/referral, barriers/challenges, patients, healthcare providers and African countries/regions	Free-text terms will be adapted to Web of Science syntax. Boolean operators, truncation and phrase searching will be used where appropriate. English language; 2011–2026.
CINAHL	STI; PN; challenges/barriers; patients and healthcare providers; Africa	Equivalent STI/STD, partner notification/referral, barriers/challenges, patient and healthcare-provider terms, and African country/region terms	CINAHL Headings and free-text terms will be used where applicable. Boolean operators will be used to combine concepts. English language; 2011–2026.

African Journals Online (AJOL)	STI; PN; challenges/barriers; Africa	“sexually transmitted infection”; “sexually transmitted disease”; STI; STD; “partner notification”; “partner referral”; “patient referral”; “provider referral”; barrier; challenge; obstacle; difficult; stigma; confidentiality; Africa and African country/region terms	Free-text searching will be conducted using combinations of the core concepts. Publication years 2011–2026.
Google Scholar	STI; PN; challenges/barriers; Africa	Combinations of terms relating to sexually transmitted infections/diseases, partner notification/referral, barriers/challenges and African settings	Google Scholar will be searched as a supplementary source. The first 200 results ranked by relevance will be screened for potential eligibility.
Reference-list searching	STI; PN; challenges/barriers	Reference lists of all included studies and relevant review articles	References will be screened to identify additional potentially eligible studies not retrieved through the electronic database searches.

frontiers
